# A Single Heterochromatin Boundary Element Imposes Position-Independent Antisilencing Activity in *Saccharomyces cerevisiae* Minichromosomes

**DOI:** 10.1371/journal.pone.0024835

**Published:** 2011-09-16

**Authors:** Sangita A. Chakraborty, Robert T. Simpson, Sergei A. Grigoryev

**Affiliations:** 1 Department of Biochemistry and Molecular Biology, College of Medicine, Pennsylvania State University, Milton S. Hershey Medical Center, Hershey, Pennsylvania, United States of America; 2 Department of Biochemistry and Molecular Biology, Eberly College of Science, Pennsylvania State University, University Park, Pennsylvania, United States of America; Duke University, United States of America

## Abstract

Chromatin boundary elements serve as cis-acting regulatory DNA signals required to protect genes from the effects of the neighboring heterochromatin. In the yeast genome, boundary elements act by establishing barriers for heterochromatin spreading and are sufficient to protect a reporter gene from transcriptional silencing when inserted between the silencer and the reporter gene. Here we dissected functional topography of silencers and boundary elements within circular minichromosomes in *Saccharomyces cerevisiae*. We found that both *HML-E* and *HML-I* silencers can efficiently repress the *URA3* reporter on a multi-copy yeast minichromosome and we further showed that two distinct heterochromatin boundary elements STAR and *TEF2*-UASrpg are able to limit the heterochromatin spreading in circular minichromosomes. In surprising contrast to what had been observed in the yeast genome, we found that in minichromosomes the heterochromatin boundary elements inhibit silencing of the reporter gene even when just one boundary element is positioned at the distal end of the *URA*3 reporter or upstream of the silencer elements. Thus the STAR and *TEF2*-UASrpg boundary elements inhibit chromatin silencing through an antisilencing activity independently of their position or orientation in *S. cerevisiae* minichromosomes rather than by creating a position-specific barrier as seen in the genome. We propose that the circular DNA topology facilitates interactions between the boundary and silencing elements in the minichromosomes.

## Introduction

The DNA in the nuclei of eukaryotic cells is packed into nucleosomes, establishing the primary level of chromatin packing. The DNA transcription, replication, recombination, and repair processes occur in the context of DNA packed into nucleosome arrays [1], [2]. The structural-functional relationship between chromatin packing and DNA transcription is manifested by segregation of nuclear chromatin into the open and active euchromatin and the condensed and repressed heterochromatin [3], [4]. The question how the transcriptionally active euchromatin is functionally separated from the inactive heterochromatin has been of considerable interest.

Previous research has focused on identifying cis-acting genetic elements termed “boundary elements” that demarcate the heterochromatin from the euchromatin [5], [6]. Such DNA elements were identified in evolutionary diverse organisms ranging from yeast to humans [7]–[9]. The heterochromatin boundary elements establish boundaries of chromatin domains by limiting the spread of silencing signals to the adjoining regions [10]. These elements are especially important when transcriptionally active genes are surrounded by condensed heterochromatin as they stop the incursion of silencing signals from the surrounding regions thereby protecting the genes from position-dependent variegation.

Similar to positional effects in higher eukaryotes, in the budding yeast *Saccharomyces cerevisiae* the telomeres and the silent mating-type loci (*HML* and *HMR*), all represent well-defined heterochromatin domains, where genes are transcriptionally silent. The transcriptionally silent copies of the mating-type genes are located at the *HML* and *HMR* silent loci near telomeres. The *HML* and *HMR* silent loci are flanked by “Essential” (E) and “Important” (I) silencer elements [11]–[14]. The *cis*-acting E and I elements are necessary and sufficient for initiating and mediating silencing in an orientation-dependent manner by interacting with a large number of trans-acting factors to repress transcription [11], [13], [15], [16]. Transcriptional repression at HM loci is a gene-nonspecific event and the silencers can repress any reporter gene [17], [18].

The silent chromatin structure does not extend indefinitely and is restricted within the HM loci and telomeres by the heterochromatin boundary elements that block silencer-mediated repression of the gene in mating-type loci as well as shield repressive positional effects of telomeric heterochromatin [7], [19]–[21]. It has been suggested that the *HML-I* silencer may itself establish a heterochromatin boundary by organizing heterochromatin in a uni-directional manner within the *HML* locus [22]. Furthermore, a tRNA gene surrounding the *HMR* locus in *S. cerevisiae* has been shown to have barrier activity and restrict the spread of silencing from the *HMR* locus [7], [8]. In addition, two special heterochromatin boundary elements, STAR and *TEF2*-UASrpg, were shown to have boundary activity in *S. cerevisiae* genome [19], [20].

In *S. cerevisiae*, the chromosomal ends contain the *X* and/or *Y*' subtelomeric repeat elements abutting the telomeric repetitive DNA. Sequences within these *X* and *Y*' subtelomeric repeats block silencing, exhibiting heterochromatin barrier activity and thus are named subtelomeric anti-silencing regions (STAR). The STAR boundary element has also been shown to counteract the silencer-driven repression of reporter genes at the *HML* locus in the genome, when interposed between the silencer and the reporter gene without transcriptional activation of the reporter [20], [23].

The *TEF2*-UASrpg located on Chromosome II in *S. cerevisiae* is an example of heterochromatin boundary element. It was identified by the silencer-blocking assay by positioning the boundary element between the silencer and the reporter gene to test its ability to counteract the silencing mechanism. The *TEF2* gene encodes the translational elongation factor-1alpha and the upstream activation sequence of *TEF2* is able to block the silencing activity and the spread of heterochromatin without transcriptional activation. In the genome, when the *TEF2*-UASrpg was placed at the *HML* locus it was able to resist transcriptional silencing of native or reporter genes in a position and orientation dependent manner [19], [24].

Distinct models have been suggested for chromatin boundary formation. In one model, boundary elements act by creating nucleosomal gaps and establishing barriers for example when placed between the silencer and the regulated gene but not upstream of the silencer or downstream of the gene [24], [25], [26]. In the other model, the boundary element could form loops reaching out to and inhibiting silencers. Within this model the boundary elements may act independently of their position versus the silencer element and are assigned to have a desilencing or anti-silencing rather than barrier activity [27]–[31]. The exact molecular mechanism may vary between distinct boundary elements and different organisms and still remains largely unknown.

In order to understand the molecular mechanism of barrier formation by STAR and *TEF2*-UASrpg, two distinct heterochromatin boundary elements, we turned to the yeast minichromosome system. Yeast minichromosomes are multi-copy circular plasmids that assemble into chromatin *in-vivo* [32] and have been used to study nucleosome positioning, chromatin remodeling, and interaction of *trans*-acting factors with *cis*-acting elements [33]–[36].

To dissect the topographic relationship of the silencers and boundary elements on a minichromosome, we generated a number of minichromosomal constructs containing different combinations of the “E” and “I” silencers from the *HML* locus with STAR and *TEF2*-UASrpg heterochromatin boundary elements. The *URA3* has been used as the reporter gene and the *TRP1* as the selection marker in all the minichromosome constructs examined. Identification of whether the *URA3* reporter gene is ON/OFF has been tested using 5-FOA biochemical selection screen in addition to growth on Uracil-deficient media.

We report here that the *URA3* reporter gene was efficiently silenced by the E and/or I silencers in the absence of heterochromatin boundary elements and the *URA3* reporter was de-repressed in the presence of STAR and *TEF2*-UASrpg elements in circular minichromosomes similar to previous studies in the yeast genome [19], [20]. However, our findings showed that the STAR and *TEF2*-UASrpg elements exhibit an antisilencing rather than boundary activity in *S. cerevisiae* minichromosomes. We propose that the topology of circular minichromosomes may help to bypass the strict positional requirements of chromatin boundaries that operate in linear chromosomes.

## Results

### Characterization of *URA3*-based reporter minichromosomes

**Figure 1:**
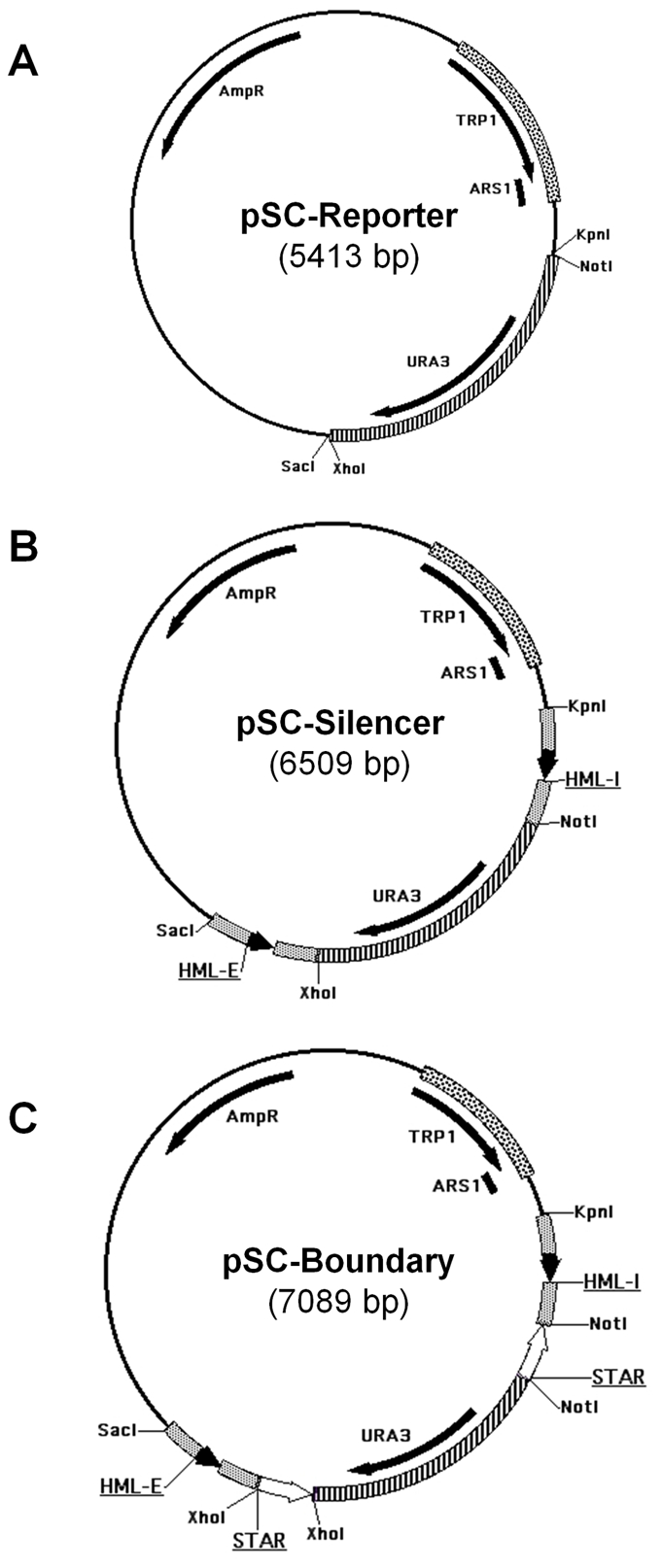
Schematic representation of the reporter, silencer, and boundary minichromosomal constructs. **A:** Physical map of the Reporter construct containing *URA3* reporter gene, *TRP1*-ARS1 for selection and propagation in *S. cerevisiae*, and *AmpR* for modifications in *E. coli*. **B:** Physical map of the Silencer construct containing the *HML -* E and I silencer elements with flanking sequences on either side of the *URA3* reporter gene. **C:** Physical map of one of the Boundary constructs. This example contains the STAR boundary element. Other boundary constructs may contain either *TEF2*-UASrpg or STAR element or both positioned either downstream or upstream of the silencing elements in different orientations (see schemes in Figures 2–6).

The minichromosome constructs generated were tested for their functionality upon transformation into *trp1-* and *ura3-* deficient yeast strains. The dependence upon *TRP1* selection marker has been used as a control for all experiments and the *URA3* served as a reporter gene for transcriptional silencing in this study (Figure 1A). Cell growth on 5-FOA [37] and inability to grow on URA- media [38] indicates that the *URA3* is repressed and therefore the cells are 5-FOA resistant, allowing us to identify whether the *URA3* reporter is silenced in the presence of silencer elements and expressed in the presence of boundary elements. Recent reports point out to a potential problem with 5-FOA screenings, due to metabolic changes caused by the 5-FOA and suggest to check the reporter gene expression for epigenetic mechanisms or heterochromatic silencing studies [39], [40]. However, in our study we have also directly assessed the *URA3* reporter gene activity in medium lacking uracil for analysis of *URA3* expression in the presence or absence of silencers and boundary elements independently of the 5-FOA assays.

**Figure 2:**
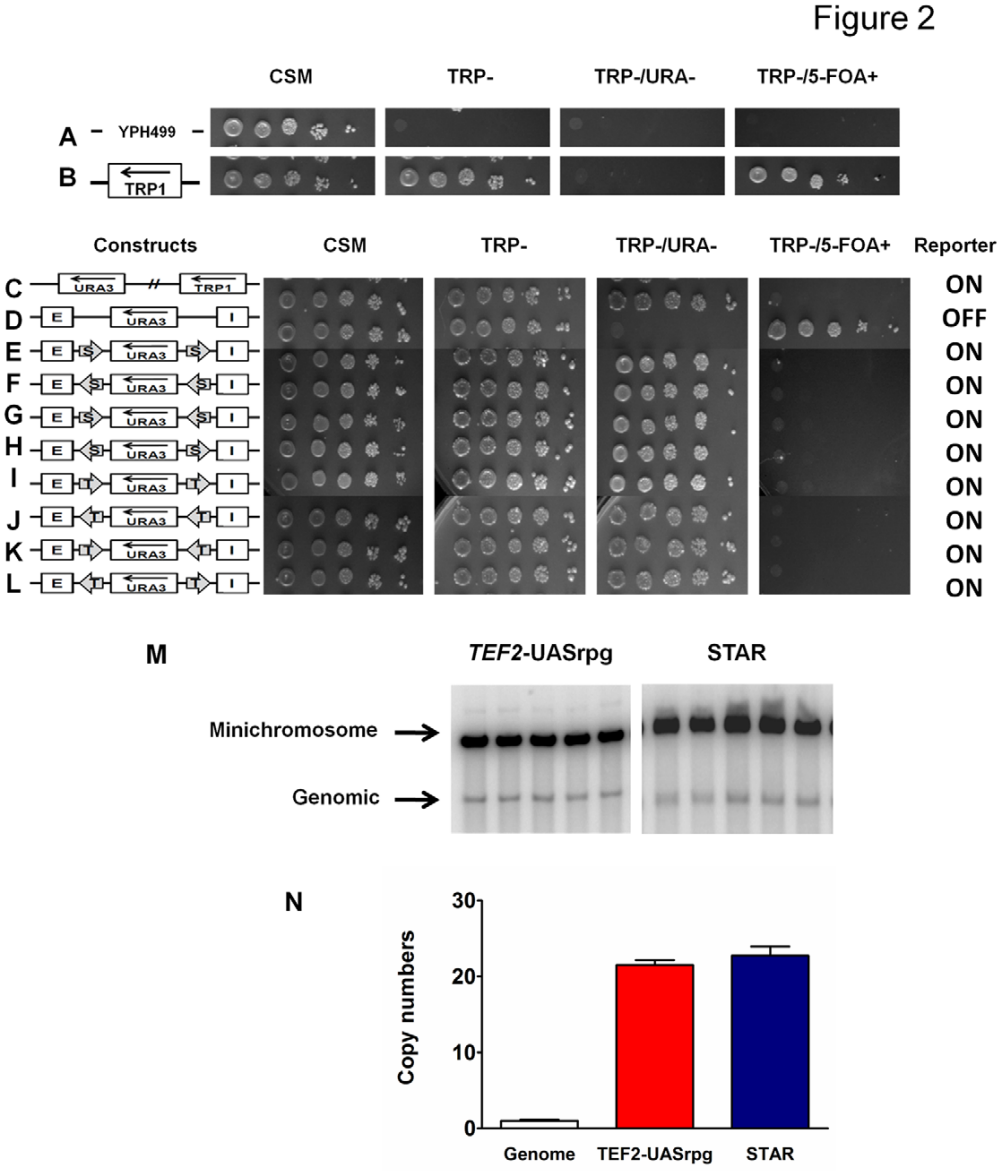
Boundary elements STAR and *TEF2*-UASrpg block the activity of the E and I silencers irrespective of their orientations. **A:** Strain YPH499 (*trp-*, *ura-*) can grow on CSM, but is unable to grow on selective media conditions [TRP-; TRP-/URA-; TRP-/5-FOA+]. **B:** Minichromosome containing the *TRP1* marker gene is able to grow on CSM; TRP-; unable to grown on TRP-/URA- (due to lack of *URA3*) and is 5-FOA resistant. **C:** Minichromosome containing both the *TRP1* marker and the *URA3* reporter genes is able to grow on CSM; TRP-; TRP-/URA- and is 5-FOA sensitive (due to the presence of the functional *URA3* product). **D:** Minichromosome containing the *HML-*E and I silencer elements is able to silence the *URA3* reporter gene and the cells are able to grow on CSM; TRP-; unable to grown on TRP-/URA- (due to lack of *URA3*) and is 5-FOA resistant. **E–L:** Two boundary elements (shown by arrows) STAR (S) and *TEF2*-UASrpg (T) were examined in different orientations in the presence of both the *HML* – E and I silencer elements. The *URA3* reporter gene expression status (on or off) was assayed by the growth phenotypes of *S. cerevisiae* cells containing various minichromosome constructs tested in different selective media by serial dilutions. **M:** Southern blot of linearized minichromosomal and genomic DNA probed with radiolabeled *TRP1*-ARS1 containing fragment. Five independent clones transformed with minichromosome construct containing *TEF2*-UASrpg (left panel) and STAR (right panel). **N:** Graphic representation of minichromosome copy numbers quantified by scanning of the Southern blots (such as shown in Figure 2M) and normalized to the genomic *TRP1*-ARS1 signal. Error bars represent Standard Deviations.

We checked the *S. cerevisiae* strain YPH499 (a-cells) for its genotype. These cells were only able to grow on complete synthetic media (CSM) without any dropouts, but could not grow on any selective media such as TRP^−^ or TRP^−^/URA^−^ or TRP^−^/5-FOA^+^ media (Figure 2A). In the presence of the *TRP1* marker, the cells were able to grow on CSM, TRP- and TRP-/5-FOA+ selective media being 5-FOA resistant, but were unable to grow on TRP-/URA- due to the absence of *URA3* gene product (Figure 2B). In the presence of both *TRP1* marker and *URA3* reporter, the cells were able to grow on CSM, TRP- and TRP-/URA- selective media, but were unable to grow on TRP-/5-FOA+ plates due to the presence of a functional *URA3* gene product the cells exhibited sensitivity to 5-FOA (Figure 2C). Efficient ten-fold serial dilutions were established to cover the range of selection from ∼2×10^7^ cells/ml to ∼2×10^3^ cells/ml in the spotting assays. The copy numbers of the multi-copy *S. cerevisiae* minichromosomes were tested throughout this work and were found to be constant for different minichromosome constructs. The numbers of minichromosomes were determined to be ∼20 copies compared to the genomic copy by southern hybridization with specific *TRP1-ARS1* probe and (Figure 2M) and quantified using the ImageQuant software (Figure 2N).

### Establishment of silencing in circular multi-copy minichromosomes

In order to study heterochromatin barrier function we had to establish robust silencing in the *S. cerevisiae* minichromosome system. Earlier reports indicate that silencing of a gene placed between the two silencer elements, *HML* – E and I, in the yeast genome is uniformly high and does not depend on the chromosomal context beyond the silenced locus [15], [22], [41].

**Figure 3 pone-0024835-g003:**
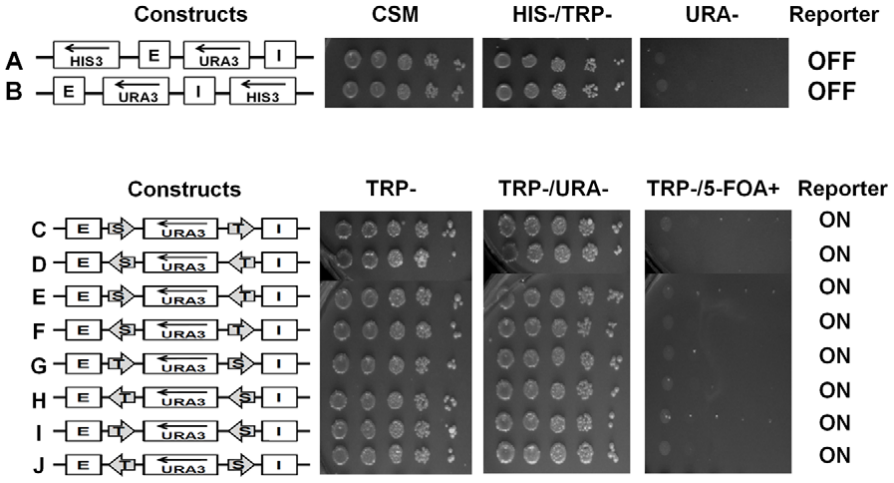
Combination of two different boundary elements blocks the silencing of the reporter from both E and I silencers. **A:** Control showing that E silencer in the minichromosome has directionality similar to the genome and only silences *URA3*, but not the *HIS3* gene placed upstream of the E silencer. These cells are able to grow on CSM and HIS-/TRP-, but are unable to grow on URA- media. **B:** Control showing that I silencer in the minichromosome also has directionality similar to the genome (like the E silencer) and only silences *URA3*, but not the *HIS3* gene placed upstream of the I silencer. These cells are able to grow on CSM and HIS-/TRP-, but are unable to grow on URA- media. **C–J:** Combination of two BE - boundary elements (shown by arrows) STAR (S) and *TEF2*-UASrpg (T) were examined in different positions and orientations in the presence of both the E and I silencers. The *URA3* reporter gene expression status (on or off) was assayed as in Figure 2.

The silencer minichromosome construct (Figure 1B) has been designed to study the effects of both the *HML* “E” and the “I” silencer elements on silencing the expression of the *URA3* reporter gene. The differences in growth phenotypes and the silencing of the *URA3* reporter gene were studied in the absence (Figure 2C) or presence of both the *HML-E* and the *HML-I* silencers placed on either side of the *URA3* reporter gene to reproduce the genomic topography as closely as possible (see schemes in Fig. 2D). Serial dilutions of the strains constructed were spotted for assaying the silencing efficiency under different selective media conditions (Figure 2C and 2D). Both *HML* – E and I silencers were capable of silencing the expression of the *URA3* reporter gene, so these cells were unable to grow on TRP-/URA- and are 5-FOA resistant (Figure 2D). Using serial dilution assay, we observed that *URA3* gene was repressed strongly enough to mimic the URA- phenotype of the control plasmid indicating that the silencers repressed the reporter gene completely. In contrast, the *TRP1* marker gene located upstream of the *HML* – I silencer was not silenced by either E or I silencer in the circular minichromosomes, apparently because both *HML* – E and I silencers have directionality in the minichromosomes as seen in the genome and only silence the expression of genes positioned in between the silencers in an orientation-dependent manner [15], [22], [41]. As an additional control we also placed *HIS3* marker gene upstream of the *HML* – E silencer and downstream of the I silencer, and we found that neither E nor I silencer were able to silence the expression of the *HIS3* gene (Figure 3A and 3B).

### STAR and *TEF2*-UASrpg inhibit silencing of the reporter on yeast minichromosomes irrespective of its orientation

We examined if the *URA3* reporter gene would be protected from silencing by two heterochromatin boundary elements, STAR and *TEF2*-UASrpg that are able to counteract the silencing in an orientation-dependent manner in the yeast genome [19], [20]. Here, we found that either two STAR elements (Figure 1C, 2E–H) or two *TEF2*-UASrpg elements (Figure 2I–L) bracketing the *URA3* reporter gene were able to inhibit the silencing by the *HML-E* and *HML-I* silencers in an orientation-independent manner in *S. cerevisiae* minichromosomes (Figure 2E–L). There were no significant differences observed in the efficiency of the cell growth on TRP-/URA- selective media and growth inhibition on TRP-/5-FOA+ media. Thus in yeast minichromosome system, unlike the genomic studies [19], [20], the two boundary elements STAR and *TEF2*-UASrpg can block both the *HML-E* and *HML-I* silencers in an orientation-independent manner.

### Combination of STAR and *TEF2*-UASrpg boundary elements on minichromosomes

To determine if two identical boundary elements are required at both ends to protect the *URA3* reporter from the silencing effects of the *HML - E* and I silencer elements we have placed STAR on one end and *TEF2*-UASrpg heterochromatin boundary element at the other end. We found that all possible combinations of STAR and *TEF2*-UASrpg boundary elements on either ends of the *URA3* in different orientations were able to protect the reporter gene from being silenced (Figure 3C–J). Thus in the context of minichromosomes two identical boundary elements at either end of the reporter gene are not required to counteract *HML-E* and *HML-I* driven silencing, and a combination of STAR and *TEF2*-UASrpg boundary elements are able to protect the *URA3* from being silenced.

### A single boundary element is sufficient to block a single silencer

**Figure 4 pone-0024835-g004:**
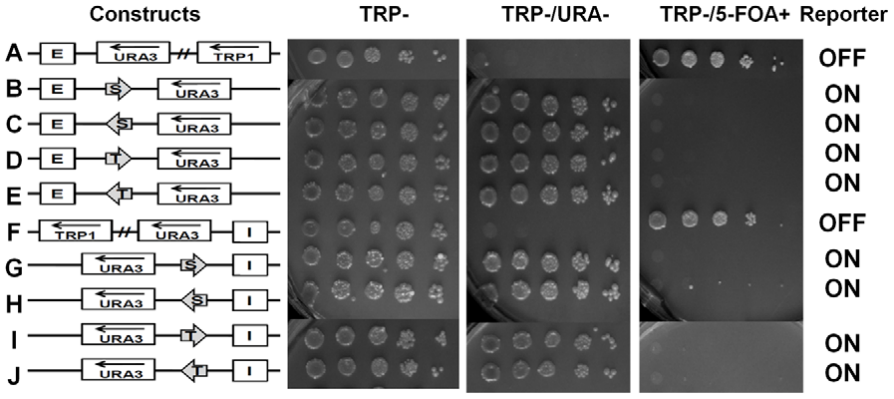
A single boundary element can counteract the silencing of the *URA3* reporter by either E or I silencer. Single BE (shown by arrows) either STAR (S) or *TEF2*-UASrpg (T) were examined in the presence of a single E silencer (A–E) or a single I silencer (F–J) in different orientations. The *URA3* reporter gene expression status (on or off) was assayed as in Figure 2.

We asked if a single silencer either E or I is sufficient to silence the expression of the *URA3* reporter gene in our minichromosome system. This was important to examine since due to limiting Sir protein concentrations (amount of silencing factors) in the yeast cells we expected a weaker silencing activity for the multi-copy minichromosomes than a single-copy silenced domain in the genome [42]. We first tested a minichromosome where only the *HML*–E silencer element has been inserted upstream of the *URA3* reporter gene (Figure 4A). We found that the *HML*–E alone was capable of silencing the expression of the *URA3* reporter gene, therefore these cells were unable to grow on TRP-/URA- plates and they are 5-FOA resistant due to the absence of the functional *URA3* gene product (Figure 4A). It has been reported earlier that in the genome, the *HML-E* and the *HML-I* silencer elements were capable of silencing alone [19], [20], [43]. By series of dilutions, we have confirmed here that the *HML-E* silencer alone was able to silence the *URA3* reporter gene on a minichromosome as efficiently as a pair of two silencers.

In another series of experiments we observed similar results with a single *HML*–I silencer element has been inserted downstream of the *URA3* reporter gene (Figure 4F). Thus the minichromosome-borne E and I silencers are both functional and efficient in silencing *URA3* reporter gene on multi-copy circular minichromosomes. Each single silencer, either E or I, is capable and sufficient to silence the reporter gene even in the absence of the other silencer in the minichromosome constructs.

Next, we examined if a single heterochromatin boundary element, either STAR or *TEF2*-UASrpg, was sufficient to block silencing imposed by *HML-E* or *HML-I* on circular minichromosomes. The boundary elements STAR or *TEF2*-UASrpg were positioned between either the *HML-E* or *HML-I* silencer elements and the *URA3* reporter. As observed in the genome, we found that a single boundary element STAR or *TEF2*-UASrpg was sufficient in blocking the silencing imposed by a single silencer either *HML-E* or *HML-I* in any orientation (Figure 4B–E and G–J).

### A single boundary is sufficient to protect *URA3* from silencing in presence of two silencer elements in circular minichromosomes

**Figure 5 pone-0024835-g005:**
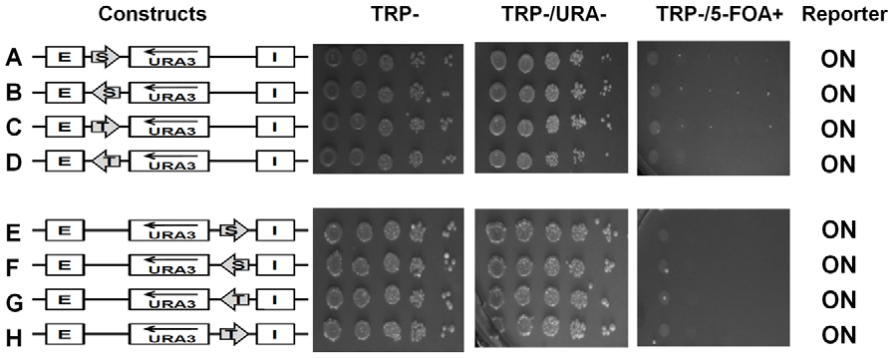
A single boundary efficiently blocks the silencing of the *URA3* reporter from both the E and I silencers. Single BE (shown by arrows) either STAR (S) or *TEF2*-UASrpg (T) were examined in the presence of both the *HML* – E and I silencer elements, placing the BE between E and *URA3* reporter (A–D) or between I and *URA3* reporter (E–H) in different orientations and the reporter gene on/off was determined by the growth phenotypes of the yeast cells tested in different selective media.

To determine if the STAR or *TEF2*-UASrpg elements behave as barriers in minichromosomes or they are able to overcome the silencing of both the E and I silencers in the presence of only one boundary element, we positioned a single boundary element downstream of either the E or the I silencer, leaving the other silencer upstream or downstream of the *URA3* reporter intact (Figure 5A–D and E–H). We found that a single boundary element was able to overcome the silencing of both the E and I silencer elements on the *URA3* reporter, even though the *URA3* was protected only from one side and there was an equal opportunity for *URA3* to be silenced by the other silencer. This finding is in striking contrast to the previous studies in linear chromosomes where the reporter gene had to be bracketed by two boundary elements to prevent the silencing in presence of both the silencers [19], [20].

### STAR and *TEF2*-UASrpg sequences are specific in blocking of silencing

**Figure 6:**
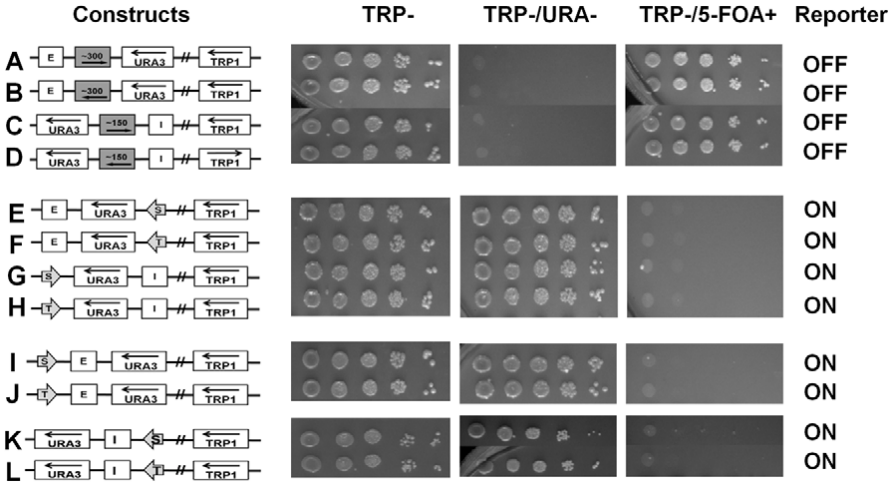
STAR or *TEF2*-UASrpg activity is sequence-specific and acts by imposing antisilencing. **A–D:** Minichromosome constructs with ∼300 bp or ∼150 bp of *Leu2* ORF sequences replacing the STAR or *TEF2*-UASrpg boundary element were positioned in between the E silencer or the I silencer and the *URA3* reporter. The E or I silencers was capable of silencing the expression of the *URA3* reporter gene. The cells were able to grow on TRP- and TRP-/5-FOA+ media being 5-FOA resistant, but unable to grow on TRP-/URA- selective media. **E–H:** Boundary elements positioned downstream of the silencer and the *URA3* reporter gene are able to block the silencing of the *URA3* reporter independent of its position unlike in the genome. These cells are able to grow on TRP- and TRP-/URA- media, but are unable to grow on TRP-/5-FOA+, as the *URA3* gene is not repressed and exhibits sensitivity to 5-FOA. **I–L:** STAR and *TEF2*-UASrpg are positioned upstream of the *HML-*E and I silencer and the *URA3* reporter gene. Unlike in the genome where the BE has to be positioned in between the reporter and the silencer, in minichromosomes the upstream BE is able to counteract silencing exhibiting antisilencing mechanism. These cells are able to grow on TRP- and TRP-/URA- media, but are unable to grow on TRP-/5-FOA+, as the *URA3* gene is not repressed and exhibits sensitivity to 5-FOA.

To confirm the specificity of the STAR and *TEF2*-UASrpg sequences in inhibiting silencing in the context of minichromosomes we used DNA sequences of similar length from the *Leu2* ORF in different orientations replacing the ∼300 bp of STAR and ∼150 bp of *TEF2*-UASrpg sequences. The control sequences inserted either between the *HML-E* or the *HML-I* silencer and the *URA3* reporter gene were unable to block the silencing mediated by the silencers and the *URA3* reporter was completely repressed by either E or I silencer elements (Figure 6A–D). Thus, blocking of the silencing activity is specific for the STAR and *TEF2*-UASrpg DNA sequences in the minichromosomes, as random sequences of similar length to STAR or *TEF2*-UASrpg were unable to protect *URA3* reporter from being silenced.

### STAR and *TEF2*-UASrpg boundary elements exhibit antisilencing activity in *S. cerevisiae* minichromosomes

To further dissect the mechanism of inhibition of silencing, we conducted a genetic test showing whether STAR and *TEF2*-UASrpg act as barriers or exhibit antisilencing activity in minichromosomes. The anti-silencing activity is defined as an ability of a boundary element to block the silencing independently of its position in relation to the silencer or the reporter distinct from the desilencing activity [28], [30]. We therefore positioned the STAR and *TEF2*-UASrpg elements downstream of the *HML-E* or the *HML-I* silencers (Figure 6E–H). We found that either STAR or *TEF2*-UASrpg was able to protect the *URA3* from being silenced even though they were not interposed between the silencer and the reporter, but was instead placed downstream of the silencers (Figure 6E–H).

Unlike the position downstream of the silencer elements, at which there is a possibility of competition between the boundary and the silencer to either activate or repress *URA3*, we next placed the STAR and *TEF2*-UASrpg upstream of the *HML-E* or the *HML-I* silencers and the *URA3* reporter gene where the silencer is in closer proximity to the reporter than the boundary. We found that in this setting the STAR and *TEF2*-UASrpg displayed a strong antisilencing activity (Figure 6I–L) as effectively as it had already been observed with positioning STAR and *TEF2*-UASrpg downstream of the silencers. Only one copy of either STAR or TEF2-UASrpg irrespective of its position in relation to the silencers or the reporter was sufficient to stop the silencing of the URA3 reporter. Thus, in contrast to linear chromosomes where the STAR or *TEF2*-UASrpg boundary elements show a position- and orientation-dependent barrier function [19], [20], in the *S. cerevisiae* minichromosomes these elements restrict silencing in a position and orientation independent manner and exhibit an antisilencing rather than barrier activity.

## Discussion

The objective of this study is creating a minichromosome model system recapitulating functional and spatial relationships between genetic elements controlling heterochromatin in yeast and facilitating its topographic analysis. We conducted a detailed characterization of STAR and *TEF2*-UASrpg heterochromatin boundary elements in minichromosomes in *S. cerevisiae* to determine if these elements act as barriers separating an active locus from silenced locus or are they able to inhibit silencing where their topology is not essential. We found that *S. cerevisiae* can maintain and pack episomal DNA into chromatin and minichromosomes at a stable copy number and thus provide a robust model for studying relationships between the heterochromatin elements and gene regulation independently of the chromosomal context. Our newly established minichromosome system can be employed as a screen for testing other candidate barriers elements such as tRNA genes and positioned nucleosomes.

We used the *HML-E* and *HML-I* silencer elements for our study, since both the E and I silencers at the *HML* locus are equally capable in silencing, unlike the silencers at the *HMR* locus [11], [17] and as the *HML* silencers have been used in previous experiments with STAR and *TEF2*-UASrpg barriers [19], [20]. In the genomic HM loci the E and I silencers are ∼3.5 Kb apart and the silencing is known to work if that distance is increased only up to a certain extent (∼6–7 Kb), after which the silencing activity decreases [7], [17], [29], [44], [45] as it is limited by the silencing propagating factors such as Sir3 [45]. In this study, in the minichromosome context, the silencing-initiating *HML*– E and I elements are ∼2 Kb apart but with ∼20 copies of the minichromosome the total DNA length through which silencing is propagated in the minichromosomes exceeds ∼10-fold the effective spreading length limit between the *HML*- E and I silencer elements in the genome. We found that the *URA3* reporter gene was completely repressed in all the ∼20 minichromosomal copies in the yeast cells, since expression of only one gene copy was sufficient for growth on URA- media as well as for inhibiting growth on 5-FOA. As the plasmid-borne silencers are less constrained than those in the genome, the silencers on minichromosomes can effectively silence the reporter genes and efficiently maintain a total length of ∼40 Kb silenced loci on minichromosomes. Similar to earlier reports, the silencing in the minichromosomes is much more robust and multi-fold higher than seen in the genome [46]. Furthermore, consistent with earlier reports [11], [47], stating that a single silencer element is capable of acting alone in the genome, we have shown for the first time that a single silencer element (either *HML* – E or I) is sufficient in silencing the *URA3* reporter gene even in circular multi-copy minichromosomes. Thus we were able to construct a circular minichromosome model system where both *HML*- E and I silencers were functional and efficient in silencing the *URA3* reporter gene in multi-copy minichromosomes in the yeast cells.

Surprisingly, in sharp contrast to the genome, where the STAR or *TEF2*-UASrpg are known to block the spreading of silencing acting as barriers, i.e. only when interposed between a silencer and the reporter gene [19], [20] with minichromosomes, we found that both STAR and *TEF2*-UASrpg were able to inhibit the silencing of *URA3* irrespective of their orientations and positions in relation to the silencer or the reporter. In minichromosomes the STAR and *TEF2*-UASrpg exhibit position-independency and antisilencing activity where only one copy of either the STAR or *TEF2*-UASrpg is sufficient in inhibiting silencing of the *URA3* reporter gene. Although the silencer elements exhibit efficient silencing of the reporter gene in a direction-dependent manner, similar to what is exhibited by silencer elements in the genome, we cannot rule out that the altered function of barrier elements on minichromosomes is (at least partially) due to the altered properties of the silencer.

**Figure 7:**
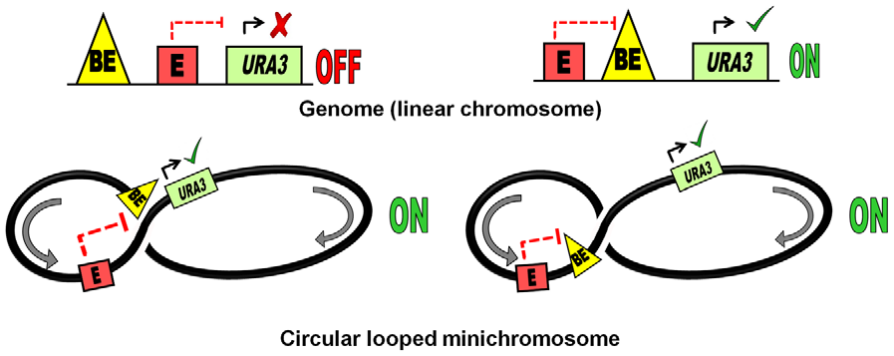
Model illustrating antisilencing activity of boundary element facilitated by minichromosome looping. **Top panels:** Within the genome, the boundary element (BE) would block silencing when located between the silencer (E) and the *URA3* gene but not upstream of the silencer. **Bottom panels:** In the minichromosomes, a close contact between the silencer (E) and the boundary element - BE (either STAR or *TEF2*-UASrpg) established by looping would prevent the silencing of the *URA3* reporter by the silencer (E) irrespective of the position of the boundary element in relation to either the silencer or the *URA3* reporter gene.

To explain the antisilencing mechanism observed in the circular minichromosomes, we propose that within a minichromosome, its circular topology would promote interactions between the boundary element and the silencer bypassing the topographical constraints (Figure 7). This would allow the boundary element to block silencing of the *URA3* reporter gene by the E silencer, irrespective of the position of the boundary element in relation to the silencer or the reporter. Thus unlike in the genome, the STAR or *TEF2*-UASrpg elements in the *S. cerevisiae* minichromosomes are capable of inhibiting the silencing of the *URA3* reporter gene by the *HML-E* and *HML-I* silencers in a position independent manner.

Why do boundary elements act so differently on a minichromosome as compared to the genome? In the genome, the silent loci are known to have an altered chromatin organization that, in addition to spreading of the silencing complex and histone deacetylation, also involves chromatin higher-order structural transitions [44], [48]–[50]. In the genome, the boundary elements need to be positioned between the silencer element and the promoter because otherwise they will be spatially hindered from the silenced region that forms a stable fold-back loop. Perhaps, this is not the case with the minichromosome where sliding of the interwound DNA helices of the covalently closed supercoiled DNA ring against each other “slithering” [51] facilitates DNA interaction at a distance and brings distal regulatory elements into close contact [52]. Furthermore, on a minichromosome associated with nucleosomes, the nucleosomal folding per se promotes contacts between the distal regulatory elements without supercoiling [53]. Looping of the telomeric heterochromatin has been shown to facilitate transcription by bringing Upstream Activating Sequences in a close contact with promoter [54] when the former was positioned downstream of the gene. We suggest that a facilitated looping may explain the more efficient functioning of the boundary elements in the minichromosome where they could find an easier access to the promoter (Figure 7) and out-compete silencers even when positioned downstream of the gene or upstream of the silencer. Based on the study of DNA minicircles excised from the yeast genome, it has been previously shown that DNA topology changes are associated with silencing [55]. Here we propose that DNA topology may regulate interactions between the boundary and silencing elements in the genome and its effect on silencing can be functionally dissected in the minichromosome system.

It is known that plasmid-borne silencers exhibit very strong silencing [46], similarly we show here that the STAR and *TEF2*-UASrpg may exhibit robust antisilencing in a minichromosomal environment. This “easy come – easy go” mode of silencing on a minichromosome implies that in this system the heterochromatin structure is relatively relaxed compared to the more rigid chromatin organization in the genome. The yeast minichromosome system that we have genetically characterized here is especially suited for isolating of the minichromosome in different functional states [32], [56] for subsequent ultrastructural analysis that may finally clarify the actual 3D chromatin organization of the silent and active minichromosomes.

## Materials and Methods

### Minichromosome Constructs

#### A) Reporter constructs:

The *URA3* reporter minichromosome construct was generated by inserting ∼1.3 kb *Fsp*I DNA fragment from the YIp5 plasmid containing ∼800 bp *URA3* reporter gene (GenBank accession number NC_001137.3, Chromosome V, 116167 to 116970). The *URA3* reporter gene (*Fsp*I fragment) is cleaved from YIp5 plasmid and inserted at the multiple cloning site (*Sma*I) of the ALT (*ARS1*, *lac*-operator, *TRP1*) plasmid. All minichromosome shuttle vectors contain an autonomously replicating sequence - *ARS1* (GenBank accession number NC_001136.10, Chromosome IV, 462354 to 463192), and a selectable marker - *TRP1* gene for selection in *S. cerevisiae* [56] (GenBank accession number NC_001136.10, Chromosome IV, 461842 to 462516) and a pBR322 vector-derived sequence with an *AmpR* gene for selection and ColE1 origin for propagation in *E. coli*. The *URA3* reporter is adjacent to the *TRP1* marker gene and in the same orientation in all the minichromosomal constructs (Figure 1A).

#### B) Silencer constructs:

The silencer constructs were generated by inserting *HML-E* and *HML-I* silencer elements into the minichromosome backbone. An ∼500 bp fragment containing the “E silencer” with ∼200 bp upstream and downstream flanking sequences (GenBank accession number NC_001135.5; chromosome III, 10966 to 11499), were PCR amplified from the *HML* locus using genomic DNA and primers with unique *Sac*I and *Xho*I restriction enzyme sites for integrating into the minichromosome (Table S1). Similarly an ∼500 bp fragment containing the “I silencer” with ∼200 bp upstream and downstream flanking sequences (GenBank accession number NC_001135.5, Chromosome III, 14364 to 14912), were PCR amplified from the *HML* locus using genomic DNA and primers with unique *Not*I and *Kpn*I restriction enzyme sites (Table S1) for integrating into the minichromosome and were verified by DNA sequencing. The PCR products were cloned into pGEMT vectors. The silencer elements were excised from the pGEMT vectors and ligated into the minichromosome constructs. The *Sac*I site in the minichromosome vector was too close to the *Xho*I site, this problem was overcome by adding a short DNA linker containing a *Sac*I site. The *HML - E* and I silencer elements have the same directionality in the minichromosome as in the genome (Figure 1B). The control *HIS3* gene (GenBank accession number NC_001147.6, Chromosome XV, 721946 to 722608) was PCR amplified from pRS413 vector (ATCC pRS series) and inserted at the *Sac*I restriction site upstream of the E silencer or at the *BamH*I restriction site upstream of the I silencer. The *HIS3* is in the same orientation as the *TRP1* and the *URA3* in the minichromosome constructs.

#### C) Boundary constructs:

The heterochromatin boundary element constructs were generated by inserting *TEF2*-UASrpg and STAR boundary elements in the minichromosome. The ∼150 bp *TEF2*-UASrpg (GenBank accession number NC_001134.8, Chromosome II, 477109 to 477257) and ∼300 bp STAR (GenBank accession number NC_001143.9, Chromosome XI, 70 to 345) were PCR amplified from genomic DNA using specific primer sets with unique restriction enzyme sites (Table S1) and verified by DNA sequencing. The PCR products were cloned into pGEMT vectors. The boundary elements were excised from the pGEMT vectors and ligated into the minichromosome constructs. The *TEF2*-UASrpg and STAR boundary elements has *Xho*I ends inserted in between the E-silencer and the *URA3*-reporter and has *Not*I ends inserted in between the I-silencer and *URA3*-reporter in the minichromosomes (Figure 1C). The STAR and *TEF2*-UASrpg were also positioned upstream of the E or the I silencer using *Sac*I and *BamH*I restriction sites. The *TEF2*-UASrpg and the STAR boundary elements have been inserted in both orientations and in different positions in the various minichromosome constructs. The control sequences of ∼300 bp (similar to STAR in length) and the ∼150 bp (similar to *TEF2*-UASrpg in length) were PCR amplified from Leu2 ORF (GenBank accession number NC_001135.5) from pRS415 vector (ATCC pRS series) and inserted at the *Xho*I or *Not*I restriction sites between the E or I silencer and the *URA3* reporter replacing STAR or *TEF2*-UASrpg boundary elements.

### Yeast strains and media

All minichromosome constructs were transformed into *E. coli* DH5α competent cells and bacterial colonies were screened using restriction enzyme digests, PCR analysis and DNA sequencing. The minichromosome constructs isolated from bacteria were re-transformed into *S. cerevisiae* a-cells YPH499 strain (MAT**a**, ade2–101°, his3-Δ200, leu2-Δ1, lys2–801^a^, trp1-Δ63, ura3–52) [57].

Yeast colonies grown on complete synthetic media lacking tryptophan (TRP-) were selected for all minichromosome constructs containing *TRP1* marker gene in the construct backbone. The functionality of the regulatory elements in various minichromosome constructs and the expression of the *URA3* reporter gene in the presence or absence of the *HML-*E and I silencer elements and STAR or *TEF2*-UASrpg boundary elements were determined using different selective media. The yeast colonies were grown on CSM (complete synthetic media, TRP- (lacking tryptophan), TRP-/URA- (lacking both tryptophan and uracil), TRP-/5-FOA+ (lacking tryptophan, but containing 5-fluro-orotic acid) and HIS- (lacking histidine).

### Southern Hybridization

The minichromosome DNA integrity and copy number were examined by Southern blotting. DNA was purified, linearized with *Xmn*I restriction enzyme digestion, subjected to electrophoretic separation on 1% agarose gel, and then transferred to Hybond-NX membrane (Amersham Biosciences), as per standard procedures [58]. The DNA was cross-linked to the membranes with UV light, and hybridized with *TRP1-ARS1* specific minichromosome probe (∼1.4 kb *EcoR*I fragment) that was gel purified and random primer labeled with [α-^32^P] dATP. After hybridization and washing the membranes were exposed to imaging screen (Bio-Rad) and the signal intensities were analyzed using Typhoon 9400 Phosphoimager (Amersham Biosciences) and quantified by the ImageQuant 5.2 software (Molecular Dynamics). The genomic hybridization signal was normalized to the size of the genomic *TRP1-ARS1* fragment recognized by the probe to determine the copy numbers.

### Spotting Assay

All yeast strains containing different minichromosomal constructs were grown to mid-log phase (A_600_ of ∼1.0) in liquid TRP- media with 2% dextrose at 30°C with aeration by shaking at 250 RPM. The a-cells not containing any minichromosome construct were grown in CSM. The optical density of all yeast cultures were adjusted to absorbance 1 at 600 nm wavelength containing ∼2×10^7^ cells/ml. Ten-fold serial dilution up to ∼2×10^3^ cells/ml of each strain was made for the spotting assay to assess *URA3* expression for assaying the silencing and insulating efficiency of the strains under different growth conditions [20], [46]. For each strain at least 6 independent transformants were verified by Southern blot analysis. Transformed cells from isolated colonies were inoculated and grown in TRP- liquid medium and spotted on to different selective media CSM, TRP-, TRP-/URA- (to check if 5-FOA resistance is due to silencing and not due to *URA3* mutation), and TRP-/5-FOA+ [Toronto Research Chemicals]. Cells with repressed *URA3* are able to form colonies in the presence of 5-FOA compound known to be toxic for cells expressing a functional *URA3* gene [38]. The selective media plates were spotted with 5 µl cells per spot and grown for 2 days at 30°C prior to imaging the plates to study the differences in growth phenotypes.

## Supporting Information

Table S1 **List of primers used in this study.**
(DOCX)
