# Supplementary material for: A Single Heterochromatin Boundary Element Imposes Position-Independent Antisilencing Activity in Saccharomyces cerevisiae Minichromosomes
Source: PLoS One. 2011 Sep 16;6(9):e24835. doi: 10.1371/journal.pone.0024835 (PMC3174977; doi:10.1371/journal.pone.0024835)
Supplement: Table S1 — List of primers used in this study. (DOCX) [file pone.0024835.s001.docx]

**Supplementary Table 1. List of primers used in this study**

| **Primers** | **Sequences** | **Used For** |
| --- | --- | --- |
| E Forward (SacI) | 5’ - AACGAGCTCGTGGATGGATCTAGGGTTTTATGC - 3’ | HML-E |
| E Reverse (XhoI) | 5’ - ACCCTCGAGGGTACGATTTTTCTGAAATCTTTGTTTACA - 3’ | HML-E |
| I Forward (NotI) | 5’ - ATTTGCGGCCGCTTTAATTATTATAAAATTATAGCAGGATAGTTC - 3’ | HML-I |
| I Reverse (KpnI) | 5’ - AGGGGTACCCCAAGAGATCGAAAGAAAGCTCCC - 3’ | HML-I |
| SacI Forward | 5’ - CACCCTTACGATTCCTTGCC - 3’ | SacI-Stuffer Fragment |
| SacI Reverse | 5’ - CCTGAGGCTGTAGCTGATGCT - 3’ | SacI-Stuffer Fragment |
| STAR Forward (XhoI) | 5’ - ACCCTCGAGGGTTTTGTTAACGTTTCAATATGGAGG - 3’ | STAR in between E silencer and *URA3* |
| STAR Reverse (XhoI) | 5’ - ACCCTCGAGGGTACTCTAACCCTATTCTAATCCAAC - 3’ | STAR in between E silencer and *URA3* |
| STAR Forward (NotI) | 5’ - ATTTGCGGCCGCTTTATTTGTTAACGTTTCAATATGGAGG - 3’ | STAR in between *URA3* and I silencer |
| STAR Reverse (NotI) | 5’ - ATTTGCGGCCGCTTTAACTCTAACCCTATTCTAATCCAAC - 3’ | STAR in between *URA3* and I silencer |
| Tef2 Forward (XhoI) | 5’ - ACCCTCGAGGGTCCGTATACTTACATATAGTAGATG - 3’ | *TEF2*Uasrpg in between E silencer and *URA3* |
| Tef2 Reverse (XhoI) | 5’ - ACCCTCGAGGGTCGCGGTCTGGGTGTATAAATGTG - 3’ | *TEF2*Uasrpg in between E silencer and *URA3* |
| Tef2 Forward (NotI) | 5’ - ATTTGCGGCCGCTTTACCGTATACTTACATATAGTAGATG - 3’ | *TEF2*Uasrpg in between *URA3* and I silencer |
| Tef2 Reverse (NotI) | 5’ - ATTTGCGGCCGCTTTACGCGGTCTGGGTGTATAAATGTG - 3’ | *TEF2*Uasrpg in between *URA3* and I silencer |
| STAR Forward (SacI) | 5’ - AACGAGCTCGTTTGTTAACGTTTCAATATGGAGG - 3’ | Upstream of E silencer |
| STAR Reverse (SacI) | 5’-AACGAGCTCGACTCTAACCCTATTCTAATCCAAC - 3’ | Upstream of E silencer |
| STAR Forward (BamHI) | 5’ - ACGGGATCCCGTTTGTTAACGTTTCAATATGGAGG - 3’ | Upstream of I silencer |
| STAR Reverse (BamHI) | 5’ - ACGGGATCCCGACTCTAACCCTATTCTAATCCAAC - 3’ | Upstream of I silencer |
| Tef2 Forward (SacI) | 5’ - AACGAGCTCGCCGTATACTTACATATAGTAGATG - 3’ | Upstream of E silencer |
| Tef2 Reverse (SacI) | 5’ - AACGAGCTCGCGCGGTCTGGGTGTATAAATGTG - 3’ | Upstream of E silencer |
| Tef2 Forward (BamHI) | 5’ - ACGGGATCCCGCCGTATACTTACATATAGTAGATG - 3’ | Upstream of I silencer |
| Tef2 Reverse (BamHI) | 5’ - ACGGGATCCCGCGCGGTCTGGGTGTATAAATGTG - 3’ | Upstream of I silencer |
| *His3* Forward (SacI) | 5’ - AACGAGCTCGCGTTTTAAGAGCTTGGTGAGC - 3’ | Upstream of E silencer |
| *His3* Reverse (SacI) | 5’ - AACGAGCTCGTCGAGTTCAAGAGAAAAAAAAAG - 3’ | Upstream of E silencer |
| *His3* Forward (BamHI) | 5’ - ACGGGATCCCGCGTTTTAAGAGCTTGGTGAGC - 3’ | Upstream of I silencer |
| *His3* Reverse (BamHI) | 5’ - ACGGGATCCCGTCGAGTTCAAGAGAAAAAAAAAG - 3’ | Upstream of I silencer |
| ~300 bp Forward (XhoI) | 5’ - ACCGCTCGAGCGGGACTAGTCAATTGTACGCCAACTTAAGACCA - 3’ | Control for STAR |
| ~300 bp Reverse (XhoI) | 5’ - ACCGCTCGAGACATGCATGCTTTATCCAAGGACCAA ATAGGCA - 3’ | Control for STAR |
| ~150 bp Forward (NotI) | 5’ - ATTTGCGGCCGCTTTGACTAGTGATGGTGATGGTGTCGCTTG - 3’ | Control for *TEF2*Uasrpg |
| ~150 bp Reverse (NotI) | 5’ - ATTTGCGGCCGCACATGCATGCCTTGAAGAGGCCAAAACATTAGC - 3’ | Control for *TEF2*Uasrpg |
